# The Kidneys and Urine

**Published:** 1843-06-24

**Authors:** 


*The Kidneys and Urine.* By J. J. Berzelius. Translated from the German, by M. H. Boyé and F. Leaming, M. D. Philadelphia: Lea & Blanchard; 8vo. pp. 179. 1843.

This small volume contains much interesting and useful matter. The study of the urine in diseases in general, is becoming every day more common, with the resuscitation of the humoral doctrines, and the advance of organic chemistry.

"It has been well ascertained that the urine contains the effete matters resulting from the nutrition and waste of the different tissues; it will, therefore, indicate any derangement in either of these processes; and as it is a secretion easily obtained, it claims much greater attention than it has hitherto received. In order to give a fuller view of the importance of the kidneys to the economy, we will state that the urine is the only true excretion, properly so called; for the perspiration may be supposed as intended rather to supply moisture to the surface, the respiration heat to the animal, and the fæces contain only the refuse or superfluous parts of the food." (Preface of the translators, p. iii.

The whole chemical history of the urine is presented here in a very small compass, and the modes of analysis, with the tests, simply and plainly detailed. We recommend the work to our readers, proposing hereafter to return to the subject, and to treat it more in detail.

